# Postharvest Practices, Perceptions, and Knowledge of Mycotoxins among Groundnut Farmers in the Adamawa, Centre, and North Regions of Cameroon

**DOI:** 10.1155/2024/5596036

**Published:** 2024-04-04

**Authors:** Pierre Germain Ntsoli, Marie Ampères Boat Bedine, Cynthia Claire Baleba, Steve Freddy Tchatcho Ngalle, Idriss Djoko Kouam, Roland Wilfried Titti, Georges Marius Etame Kossi, Aoudou Yaouba

**Affiliations:** ^1^Phytopathology and Agricultural Zoology Research Unit, Department of Agriculture, Faculty of Agronomy and Agricultural Sciences, University of Dschang, P.O. Box 222, Dschang, Cameroon; ^2^Institute of Agricultural Research for Development, Agricultural Research Center (ARC), Wakwa, P.O. Box 65, Ngaoundere, Cameroon; ^3^Genetics, Biotechnology, Agriculture and Plant Production Research Unit, Department of Agriculture, Faculty of Agronomy and Agricultural Sciences, University of Dschang, P.O. Box 222, Dschang, Cameroon

## Abstract

In many parts of the world, including Cameroon, mycotoxin contamination of groundnuts remains a major constraint affecting their use as food. Understanding the contributing factors is an essential intervention to reduce contamination and people's exposure to these harmful toxins. The aim of this research was to identify the factors associated with the knowledge, perceptions, and postharvest practices of groundnut farmers in three production basins in Cameroon. Data were collected through surveys and analyzed using descriptive methods and logistic regression models. The results show that groundnut farmers are little aware of the existence of mycotoxins (12%) and totally unaware of the existence of aflatoxins (100%). Only 7.1% of these farmers are aware of the effects of mycotoxins on consumer health. After evaluation, the large majority of farmers scored poor marks for knowledge (86%) and practice (98.7%) in the management of mould and mycotoxins in groundnuts. Knowledge of mycotoxins was positively associated with the level of education [OR = 3.42; (95%-IC: 1.00–16.00); *p* < 0.05] and region [OR = 4.49; (95%-IC: 1.09–20.3); *p* < 0.05]. Farmers' good practices were linked to their production experience [OR = 6.06, (95% CI: 0.91-18.4), *p* = 0.035]. The use of mouldy groundnut for feed was associated with age [OR = 3.34, (95% CI: 1.14–10.2), *p* = 0.03], sex [OR = 0. 43, (IC-95%: 1.14–1.05), *p* = 0.026], marital status [OR = 0.35, (IC-95%: 0.14–0.79), *p* = 0.015], and production region [OR = 0.27, (IC-95%: 0.13–0.56)]. In conclusion, groundnut farmers had insufficient knowledge of mycotoxins, no knowledge of aflatoxins, and suboptimal handling and storage practices for this commodity. This contributes to increasing the risk of exposure for the population and requires mitigation measures, including awareness campaigns on mycotoxins, specifically aflatoxins, and capacity building for farmers in terms of storage and postharvest management of foodstuffs.

## 1. Introduction

Of all oilseed crops, groundnuts are likely to make a major contribution to food security. Its supply or richness in proteins and lipids makes it a major asset for the world's 7 billion inhabitants [1–3]. Groundnuts are essential because of their major involvement in many industrial processes, but they are also, to a lesser extent, a source of income and a key element in the subsistence of the populations of sub-Saharan Africa [4–7]. Over the centuries, its cultivation has spread far beyond its original range in South America [8]. In the twenty-first century, global groundnut production between 2012 and 2022 has fluctuated between 35.5 and 47 million tonnes, with a land area of between 20 and 25.4 million hectares. This represents an increase of 32.3% in production and 27% in the cultivated area over the decade [9].

In Central Africa, Cameroon has the unique distinction of being considered the mother country of the subregion because of its gross domestic product (GDP), which is strongly associated with agriculture. In this country, groundnuts are both a staple food and cash crop, and an essential component of many traditional and cultural household meals [10]. However, in the current production context, the groundnut sector is adrift and facing a number of technical constraints, forcing farmers to relegate it to the status of a rotation crop. In addition, Hamasselbé [10] and Chotangui et al. [11] described the Cameroon groundnut sector as being prone to soil impoverishment and degradation, leaf diseases (leaf spot and rust), pod predators before and after harvest, climate change (insufficient and erratic rainfall), the scarcity of improved varieties for local farmers, and the inadequacy of postharvest technologies. One of the consequences of these facts is the contamination of groundnuts by mycotoxins, including aflatoxins [12–15], which are, compared with synthetic pollutants, food additives, and pesticide residues, the most important risk factor in food [16]. However, chronic exposure to aflatoxins has been linked to liver cancer, reduced immunity, and the progression of diseases such as hepatitis B and HIV/AIDS, as well as stunted growth in children. Even more seriously, acute exposure to excessively high levels of aflatoxins can lead to death [17–19], as reported in Kenya in 2005 [20].

Although no cases of aflatoxicosis have yet been officially reported in Cameroon, publications on groundnuts widely consumed by rural and urban populations report high levels of aflatoxin contamination in this country. Evidence for this includes multimycotoxin work by Abia et al. [21] who reported that groundnuts and their by-products intended for human consumption were the most concentrated in aflatoxin B1 with an overall average level of 47–52 *μ*g·kg^−1^. Similarly, Kana et al. [22] reported the levels of total aflatoxin contamination ranging from 39 to 950 *μ*g·kg^−1^ in local groundnut meal intended for animal feed. The conclusion drawn from previous studies is that, firstly, groundnuts produced in Cameroon are not free of aflatoxins, and secondly, contamination levels in peanuts produced in Cameroon are well above the limit set by the European Union (EU) or the Food and Drug Administration (FDA). This proves that people are exposed to these harmful toxins and are, therefore, prone to food insecurity and potentially serious public health problems. It is also important to mention that groundnut production in Cameroon is heavily dependent on local smallholders, who provide most of the supply of this essential foodstuff. All of the above points highlight the inability of these smallholders to produce aflatoxin-free groundnuts. This hypothesis is supported in Africa in general by Wagacha and Muthomi [23], Chauhan and Chauhan [24], and Jallow et al. [25].

In view of this, it is imperative to take action to alleviate the current situation and the risk of exposure for consumers. The potential economic losses and risks resulting from aflatoxin contamination can be reduced by promoting the dissemination of knowledge on the subject and raising awareness of postharvest handling of groundnuts [26]. More than ever, the latter can be seen as the first step towards identifying and designing mitigation measures [27]. However, in the case of Cameroon, the levels of awareness, knowledge, and perceptions of smallholder groundnut farmers regarding mycotoxins, specifically aflatoxins, as well as their postharvest management of this foodstuff, are not really known. In the light of all the above, the present study complements all those carried out on the quantification of aflatoxin content in groundnuts, with a view to establishing a diagnosis that will make it possible to understand the intrinsic and extrinsic factors linked to the high levels of contamination observed in the past. To this end, a questionnaire was designed to assess the factors that determine the contamination of groundnuts by aflatoxins in Cameroon by highlighting the awareness, knowledge, perceptions, and postharvest practices of smallholder groundnut farmers in Cameroon with regard to contamination by mould and aflatoxins.

## 2. Materials and Methods

### 2.1. Study Area

The study population was located in the Republic of Cameroon, in the main groundnut-producing regions, situated in different agroecological zones (AEZs), namely, the Adamawa region (Mbé) located in the Guinean savannah zone (7°20′–8°30′N), the Centre region (Bafia and Ombessa) located in the bimodal rainfall forest zone (3°58′–5°00′N), and the North region (Gaschiga and Ngong) located in the Sudano-Sahelian savannah zone (8°25′–11°00′N). The bioclimatic and geographical characteristics of the regions surveyed are presented in Table 1.

### 2.2. Survey Design, Population, and Sampling

The number of groundnut farmers to be surveyed was determined using the normal approximation of the following binomial distribution [30] equation:

(1) $$\begin{array}{c} n=\frac{U_{1-\propto /2}^{2}\;x\;p\left(1-p\right)}{d^{2}}, \end{array}$$

where *n* is the number of groundnut farmers surveyed; *U*_(1 − *α*/2)^2^_ = 1.96 is the quantile of a standard normal distribution with a probability value of 0.05; *p*=0.50 is the proportion of the population of groundnut farmers; and *d* is the expected margin of error of any parameter to be calculated from the survey. For the present study, the expected margin of error (d) is set at 0.05 (this value is close to zero to obtain an accurate estimate of the parameters) because the actual population of groundnut farmers is unknown. The sample size obtained from equation (1) is equal to 384.15 groundnut farmers to be surveyed.

A multistage random sampling method described by Sukhatme and Sukhatme [31] was followed for sampling respondents. At the first stage, five subdivisions in Cameroon were selectively chosen: Bafia and Ombessa (Centre region), Gaschiga and Ngong (North region), and Mbé (Adamawa region). This choice was based on the intervention zones of the Groundnut Germplasm Project (GGP), the areas under cultivation, national groundnut production statistics, ease of access, and the need for good coverage of the country. At the second stage, five villages were randomly selected from each selected subdivision. At the third, at least ten groundnut farmers were selected in each village by systematic random sampling, creating a total of 384 respondents consistent with the sample size obtained from equation (1).

### 2.3. Tool for Data Collection and Procedure

The surveys were carried out between May and September 2022 and targeted farmers who had sown, harvested, and stored groundnuts during the actual growing season (long wet season). After presenting the research objectives to the farmers and obtaining their consent, data were collected using a semistructured questionnaire with open and closed questions. The questions included qualitative and quantitative data focusing on the socioeconomic characteristics of the respondents, their postharvest practices, and their knowledge and perceptions of the contamination of groundnut kernels by moulds (*Aspergillus flavus*) and mycotoxins (especially aflatoxins). The questionnaire was digitized and downloaded onto a Kobo Toolbox server, and deployed and administered via manipulated devices (Android tablets).

To assess knowledge and practice, the method described by Ul Haq et al. [32] was employed in this study. This method assigns a value of one point (01) to each correct answer, while incorrect answers and answers where the respondent knows nothing are assigned a point value of 0. Knowledge and practice scores for each respondent were calculated by summing the scores obtained for each question, and the overall score was classified as good or poor, following the approach used by Ul Haq et al. [32] with some minor modifications. Thus, farmers with a score of less than 60% correct answers were classified as having poor knowledge or practice. Sixteen questions were asked about knowledge, and the scores, therefore, ranged from 0 to 16. Regarding the postharvest practices of farmers, 12 questions were asked and the total score varied from 0 to 12 per individual.

### 2.4. Data Analysis

All data were downloaded from the Kobo Toolbox server in the form of an Excel file, processed, transferred, and then analyzed using R software version 4.2.3 [33]. The result was a data matrix of 380 rows representing groundnut farmers, as 4 survey forms were deleted after processing the database due to missing data. Data on farmers' sociodemographic and economic characteristics, knowledge and perceptions of moulds, mycotoxins, particularly aflatoxins, and postharvest groundnut storage were analyzed using Pearson's chi-square test, Fischer's exact test and ANOVA following the approach proposed by Sjoberg et al. [34]. The significance level was set at 5%, and the means were separated by Tukey's test.

Binary logistic regression was used to determine the factors associated with knowledge of mycotoxins, knowledge and practice scores, and agronomic practices used by farmers. Models containing the explanatory variables were built using the gtsummary package [34], and the variance inflation factor (VIF) was examined for each variable using the car package [35] to measure collinearity. If 0 < FIV < 5, there was no evidence of multicollinearity. If 5 ≤ FIV ≤ 10, there is moderate multicollinearity, and finally, if FIV > 10, there is strong multicollinearity between predictors [36, 37]. The degree of susceptibility (probability) of knowing about mycotoxins according to the selected factors was measured by calculating odds ratios. Variables with a significant effect in the final model were identified using a global test on the final model represented by the following formula:

(2) $$\begin{array}{c} ln\;\left[\frac{\pi \left(x\right)}{1-\;\pi \left(x\right)}\;\right]=\alpha_{0}+\alpha_{1}x_{1}+\alpha_{2}x_{2}+...+\alpha_{n}\alpha_{n}+Ɛ. \end{array}$$

## 3. Results

### 3.1. Sociodemographic Characteristics of the Communities Surveyed

The sociodemographic characteristics of the study participants are presented in Table 2. The majority of farmers were small-scale farmers who depended essentially on farming to meet their primary needs. Respondents ranged in age from 18 to 90 years, with almost half (48%) aged between 30 and 45 years. Half of the respondents (50.3%) were women as shown in Table 2. Around 77.6% had at least some formal education, indicating a certain level of literacy among farmers. The majority of farmers surveyed farmed land areas of less than or equal to 0.5 ha (56.3%) and only 18.4% farmed areas greater than or equal to 1 ha, with 79.5% of their groundnut production destined simultaneously for self-consumption and sale, for an average income of 636 600 FCFA ($1043.7) per groundnut season. All sociodemographic characteristics (gender, age of respondent, marital status, level of education, land area under groundnut production) were significantly different in the three administrative districts.

### 3.2. Postharvest Practices of Groundnut Farmers in Terms of Mould and Mycotoxin Management

The postharvest practices of groundnut farmers are presented in Table 3. After harvesting as shown in this table, all farmers sun-dry the pods (100%), generally over a period of seven days (59%), although drying times longer than seven days are common in the Adamawa (63%) and North (85%) regions. Drying is carried out mainly at home (96%), either on the floor (58%), on a tarpaulin (36%), or on a cemented area (6.2%). None of the farmers used a moisture meter to check the quality of the drying process and assess the moisture content of the groundnuts. Instead, they use traditional or empirical methods such as the sound produced by the pods (85%) and touch by appreciating the ease with which the pod cracks (41%). Before storage, a large proportion of farmers (87%) sort the pods. The pods are then stored for up to 6 months (69%), very often in polypropylene (75%), polyethylene (14%), and jute (5.2%) or airtight (4.7%) bags. The main indicators of groundnut deterioration known to farmers are colors other than those specific to the grain (77%), taste/bitterness (29%), and insect damage (29%). More than half of the farmers use manual threshing methods (68%) to shell the groundnut pods, although there are a high proportion of farmers using mechanical threshers in the Adamawa region (70%). In general, the postharvest practices of the majority (98.7%) of farmers were poor and varied between the three regions. No relationship of dependence was established between the practice scores obtained by farmers and the region (*P*=0.17); farmers in Adamawa (99%), Centre (98%), and North (100%) had approximately the same poor practice scores for mould and mycotoxin management (Table 3).

**Figure 1:**
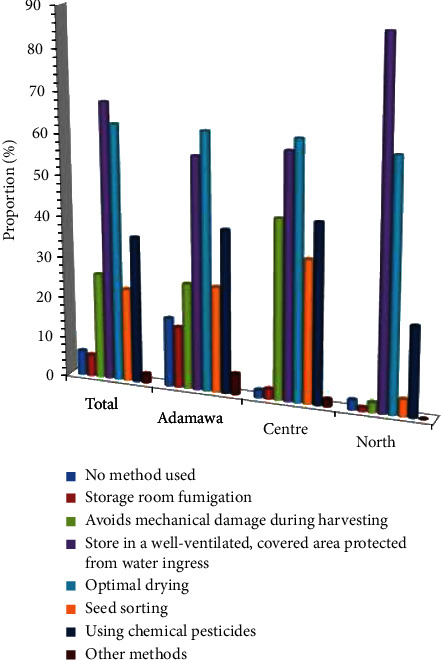
Methods used to ensure that grains are well maintained before storage.

**Figure 2:**
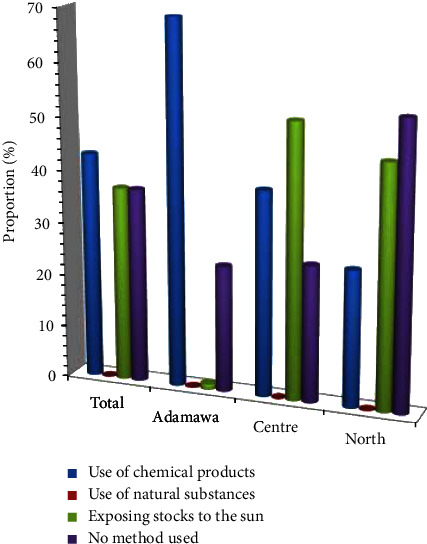
Methods used to deal with constraint storage.

Aware of the potential damage that can occur during storage, farmers use different methods to ensure that the seeds are well preserved. The methods used by farmers to ensure that grains are well preserved before being put into storage are shown in Figure 1. As shown in this figure, most farmers ensure that the grains are dried as much as possible (63%), store them in well-ventilated, covered granaries protected from seepage (68%), and use synthetic products by sprinkling the bags before storing them (36%). Only a small proportion of farmers fumigated their rooms before storage (5.3%) (Figure 1). Similarly, to deal with constraints during storage, farmers use chemical products (43%) and expose stocks to the sun (37%), while some remain indifferent to these constraints (37%), as shown in Figure 2. None of the farmers admitted to using natural products or substances during storage (Figure 2).

### 3.3. Farmers' Perceptions of Groundnut Kernel Quality

**Figure 3:**
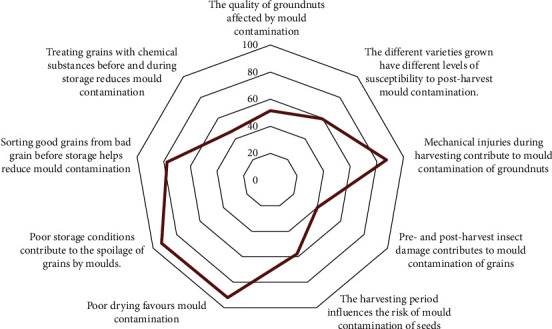
Groundnut farmers' perception of grain quality.

Although groundnuts are spoiled, only 51.5% of farmers consider them to be of poor quality and unfit for human or animal consumption. Their perceptions led them to attribute the deterioration of the grain to inadequate drying (92.1%) and poor storage conditions (92.8%), generally in damp places. In addition, farmers are able to perceive mechanical damage during harvest (87.2%), insect damage in the field and in storage (40.1%), grain sorting (77.5%), the level of susceptibility of the varieties used (59.5%), the harvesting period (57.7%), and the chemical treatment of the grain before storage (46.5%) as reasons that can increase or reduce mould contamination and postharvest losses during storage (Figure 3). Regarding varieties, some farmers themselves stated that “The Manipintar variety is more susceptible to mould contamination during storage than all the other varieties grown, but they continue to produce it because of its high yield.”

### 3.4. Farmers' Knowledge of Mycotoxin and Aflatoxin Contamination in Groundnuts

Farmers' knowledge of mycotoxins and aflatoxins is shown in Table 4. Of the 380 respondents, the majority (86%) were in the poor knowledge category. No dependency was found between the knowledge level or score and region (*χ*^2^ = 2.433, *P*=0.3), with farmers in Adamawa (89%), Centre (86%), and North (82%) having approximately the same (poor) knowledge scores for moulds and mycotoxins. Of the 380 farmers surveyed, less than half (12%) had ever heard of mycotoxins, none (0%) had ever heard of the term aflatoxin, and only 7.1% and 7%, respectively, were aware of the link between mould and mycotoxin contamination of kernel in groundnuts, and the potential effects on consumer health. Farmers in the Centre region (22%) were more aware (*χ*^2^ = 28.349, *P* < 0.001) of mycotoxins than those in Adamawa (8.1%) and the North (1.7%) as shown in Table 4.

**Figure 4:**
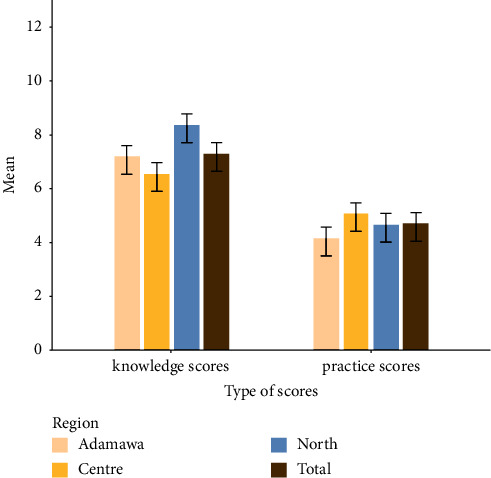
Average scores for farmers' knowledge, agronomic, and postharvest practices concerning mycotoxins in the Adamawa, Centre, and North Cameroon regions. Data are means ± standard deviation, *p* < 0.05 significant by the analysis of variance (ANOVA).

Overall, the mean score values for knowledge and practices are presented in Figure 4. The mean mycotoxin knowledge score among study respondents was 7.27 ± 2.57, out of a possible 17, indicating a low level of knowledge among groundnut farmers. Regarding the farmers' practice score, their mean practice score for groundnut farmers was 4.18 ± 1.2, out of a possible 12 points. No significant differences were observed for the farmers' practice score when comparing the three study regions, but significant differences were observed for the knowledge score (Figure 4).

### 3.5. Factors Associated with Farmers' Knowledge and Practices concerning Mycotoxins

The sociodemographic factors associated with knowledge of mycotoxins are presented in Table 5. Groundnut farmers' knowledge of mycotoxin contamination of groundnuts was positively related to the level of education [OR = 3.42 (95% CI: 1–16), *p*=0.073] and region [OR = 4.49, (95% CI: 1.09–20.3), *p*=0.044]. Thus, the knowledge of mycotoxins among farmers with secondary education was 3.42 times higher than among farmers with no education. Similarly, farmers in the Centre region were 4.49 times better informed about mycotoxins than farmers in the Adamawa region. Age, gender, marital status, and years of experience in cultivation were not associated with farmers' knowledge of mycotoxins as shown in Table 5, in the column concerning farmers' knowledge. Concerning the use of mould-contaminated grain as the food source (human or animal), the logistic regression model highlights a behavioral association with age [OR = 3.34 (IC-95%: 1.14–10.2), *p*=0.03], gender [OR = 0.43, (IC-95%: 1.14–1.05), *p*=0.026], marital status [OR = 0.35, (IC-95%: 0.14–0.79), *p*=0.015], and production region.

The associations between the sociodemographic characteristics of farmers and their knowledge and practice scores are presented in Table 6. The knowledge scores (good or poor) obtained by the farmers are associated with the production region [OR = 3.31 (IC-95%: 0.05–1.47), *p*=0.015], while the practice scores are influenced by the number of years of experience in cultivation [OR = 6.06, (IC-95%: 0.91–18.4), *p*=0.035]. Thus, farmers with more than twenty years of experience in the crop have practice scores 6 times higher than those of farmers with less than ten years in groundnut production. Similarly, the knowledge scores of farmers in North Cameroon were 3.31 higher than those of farmers in Adamawa (Table 6).

## 4. Discussion

Mycotoxin contamination of foodstuffs remains a major public health problem in many parts of the world, particularly in underdeveloped countries [20, 21, 38], where the knowledge, awareness, and practices of rural subsistence farmers regarding mould contamination of food crops remain poorly studied [39]. In the case of Cameroon, research concerning the presence of mycotoxins is encouraging, and the few local studies aimed at making up for the lack of information on this subject of public interest [21, 22, 40–45]. However, these are limited to their quantification in foodstuffs, without identifying the potential causes associated with these levels of contamination, which are very often above the standards set by the FDA (Food and Drug Administration), JEFCA (Joint FAO/WHO Expert Committee on Food Additives), and the European Union (EU). The survey was conducted to assess the knowledge, perceptions, and practices of groundnut farmers in Cameroon regarding the contamination of groundnut grains by moulds and mycotoxins (particularly aflatoxins) and to identify the factors associated with farmers' knowledge and management methods. It is important to note that 90% of the national groundnut supply comes from the communities involved in its production. The importance of groundnuts in people's diets has been demonstrated, with several farmers saying, “I use groundnuts in many cooking sauces based on okra, foléré, vernonia, and cassava leaves.”

In the current study, it was found that the postharvest practices used by groundnut farmers are variable and depend on the survey area. These practices help to minimize or increase the risk of spoilage and mycotoxin contamination of groundnuts, particularly aflatoxins, as has been pointed out in numerous studies [14, 17, 46–49]. Overall, many suboptimal postharvest practices were identified among the groundnut farmers surveyed across Adamawa, Centre, and North Cameroon. Similar observations highlighting poor postharvest practices have been made in many other studies in other African [47, 48, 50–53] and Asian countries [54–56], indicating that it is a global problem. For example, optimal pod drying is one of the postharvest methods most widely used by groundnut farmers. However, the limitation associated with the effectiveness of this method is that all the groundnut farmers interviewed said that they used empirical or traditional methods to confirm that the drying was effective and that the kernels were dry enough. A similar observation was made among caregivers in Kenya by Lesuuda et al. [51].

Similarly, several farmers have admitted that they prefer to use synthetic pesticides rather than natural products. While their effectiveness is undeniable, there is a downside to using them to preserve grains, notably the risk of ingesting pesticide residues. A recent study by Maptue et al. [7] highlights the effectiveness of formulations based on biochar and essential oils in preserving groundnuts, which constitutes an important basis for biocontrol and improved storage of this commodity. On the other hand, storage structures and the type of bag used play a vital role in mitigating the risks of mycotoxin contamination in the postharvest phase [57, 58]. Another striking finding of our study is that very few groundnut farmers use modern storage facilities. In addition to this weakness in storage structures, many farmers store their groundnut crops in polypropylene bags, regardless of the region. However, the structure of these bags means that they are not airtight [49], which facilitates fungal contamination and the synthesis of aflatoxins [52, 59].

With regard to farmers' knowledge, the results of this study indicate that only 12% and 7% of groundnut farmers surveyed were aware of the existence of mycotoxins and their effects on health, respectively, and that no farmer was aware of aflatoxins. In addition, farmers in the Centre region (22%) were more aware of mycotoxin contamination of kernels than those in Adamawa (8.1%) and the North (1.7%). These results are similar to those of Parimi et al. [60], who after a survey of groundnut farmers in India reported that only 1.4% of respondents had heard of aflatoxins. In Vietnam, only 10.2% of maize farmers were aware of aflatoxins [61]. Moreover, through a survey of spice retailers in Tanzania, Fundikira et al. [62] indicated that a very small proportion (3.3%) of respondents had actually heard of aflatoxins and were aware of aflatoxin contamination of spices during storage. In Ghana, 92% of the participants had never heard of aflatoxins, yet 62% of them stated that they generally observed the formation of moulds in their cereals/grains [63].

Nevertheless, our results are contrary to those of other studies carried out across Africa. For example, Kortei et al. [44] reported that around 50% of respondents to their surveys across twelve regions of Ghana were aware of aflatoxins. In Kenya, Marechera and Ndwiga [64] reported a relatively high level of awareness and vigilance (>90%) with regard to aflatoxins in regions where cases of aflatoxicosis have led to the death of populations. In the latter case, the aflatoxicosis-related events that led to the deaths of several people in Kenya did much to raise public awareness of the danger posed by aflatoxins [65]. Based on other studies related to cases of mycotoxicosis, Stepman [66] concluded that most African countries have a high level of knowledge in areas where epidemics have occurred in the past. However, no case of an epidemic related to mycotoxins has yet been officially reported in Cameroon, which explains, among other things, why the population is less aware and conscious of the existence of mycotoxins. Consequently, the level of awareness and knowledge of populations in relation to mycotoxins is a function of the large-scale occurrence of mycotoxicosis events or tragedies within an area, region, or nation [61].

One remarkable finding was that spoiled groundnut kernels are still used for human and/or animal consumption, and some farmers stated that “Even mouldy groundnuts, I consume them in my households.” Similar observations were made by Magembe et al. [26] in the Kilosa district of Tanzania, and by Beyene et al. [67] and Bereka et al. [68] in Ethiopia. In Ghana, Kortei et al. [69] reported that sorghum with visible mould was used to prepare local alcoholic beverages with impunity, which certainly led to the consumption of mycotoxins. However, farmers are aware that spoiled groundnuts are of poor quality. This use of spoiled kernels for food is simply the result of a lack of knowledge about mycotoxins and their effects on health [70], which is one of the most important factors in people's exposure to these harmful secondary metabolites. In such a case, the urgency of disseminating information on the potential danger must be rigorously discussed with the players in the sector and consumers.

Farmers' knowledge of mycotoxins was influenced by their socioeconomic and demographic characteristics. Factors that help explain groundnut farmers' knowledge of mycotoxins in Cameroon include the level of education and production area. These results are in line with those obtained by Njeru et al. [71] and Lesuuda et al. [51] who worked in Kenya. Knowledge of mycotoxins is positively related to the level of education [27, 72]. According to Dosman et al. [73], people with a high level of education are likely to be better informed and, consequently, more aware of certain types of risks associated with food additives or pesticides than people with a lower level of education. Furthermore, our results show that neither age, sex, marital status, nor income influences groundnut farmers' knowledge of mycotoxins in Cameroon. These results seem to contradict those obtained by Magembe et al. [26] in a survey conducted in the Kilosa district of Tanzania. This can be explained by the absence of an awareness campaign concerning the contamination of foodstuffs by mycotoxins. In fact, in the absence of a public awareness campaign on mycotoxins, the only effective means of accessing information on mycotoxins is through education. Mycotoxin awareness campaigns using effective means of dissemination have the capacity to reach a wide range of people within a population regardless of their sociodemographic characteristics. Strosnider et al. [20] justify this using unpublished data from the CDC (Centres for Disease Control and Prevention), which found that during the 2005 epidemic in Kenya, people who received information on the importance of drying and storing maize as part of an aflatoxin awareness campaign run by the FAO and the Kenyan Ministries of Health and Agriculture had lower serum aflatoxin levels than those who did not receive this information. Farmers' awareness of mycotoxins will influence their ability to adopt good farming practices and new technologies. This is justified by the work of Johnson et al. [74], who were able to show that due to their knowledge of mycotoxins, smallholder maize farmers in Nigeria were more susceptible to adopt Aflasafe for aflatoxin biocontrol.

In this study, the majority of groundnut farmers scored poorly (below 60% correct answers) in knowledge (86%) and practice (98.7%) regarding the management of grain contamination by moulds and mycotoxins. In Kenya, 61.8% and 75.4% of the childcare workers surveyed presented poor knowledge and practice scores, respectively, concerning aflatoxin and fumonisin contamination of sorghum [51]. The management methods or practices employed by farmers are largely influenced by their knowledge and perceptions [75], which, in turn, will have an impact on the nutritional and health quality of the final product, and on its use despite its quality. The resulting poor preharvest and postharvest practices are, therefore, the main factors that promote mycotoxin contamination [26, 76–78]. In the case of mould and mycotoxin management by farmers, unawareness of the existence of mycotoxins naturally translates into low levels of knowledge and poor practice (pre- and postharvest) in managing mycotoxin contamination. Kumar and Popat [56] concluded that due to their high level of ignorance, farmers have neglected certain aspects of preharvest and postharvest pod management. This lack of management is problematic, especially when it is known that groundnut is a suitable substrate for the growth of the aflatoxin-producing fungi, *Aspergillus flavus* and Aspergillus parasiticus [79, 80]. In the present study, this is more demonstrable and understandable through the practices recorded during the survey (Table 3). In the postharvest phase, for example, most farmers dry their groundnuts on the ground. To check the moisture content of the grain and the effectiveness of the drying process, none of them used a moisture meter, preferring traditional methods such as sounding the pods. These practices are inadequate and can lead to pods with a high moisture content (>8%) being stored [51, 81, 82]. Hermetic bags are rarely used for storage. However, Navarro et al. [83] have demonstrated their effectiveness in limiting the aflatoxin contamination of groundnut kernels during storage. In addition to limiting mycotoxins, these practices are essential for limiting postharvest losses, which are very often the cause of major economic losses and food insecurity around the world, particularly in Africa [84]. It is important to mention that all practices important for mitigation are more influenced by the fact of being aware of the existence of mycotoxins.

Among the factors explaining the scores obtained, the experience of the farmers in groundnut cultivation was not associated (*P* > 0.05) with the knowledge scores but was positively associated with the practice scores obtained by them (Table 6). This could be explained by the fact that the most experienced farmers are aware that poor practices in the field and at the postharvest phase promote the development of moulds on the kernels. The vast majority of farmers were aware of mould and its impact on grain quality, but only a very few proportions (7.1%) were aware of the link between mycotoxins and mould. Yet the presence of mycotoxins in foodstuffs depends, firstly, on the growth and survival of mycotoxinogenic fungi on or in the foodstuffs and, secondly, on the environmental conditions suitable to the synthesis of mycotoxins [17]. Knowledge scores were also found to be positively associated with the production area. Groundnut farmers in the North region had higher knowledge than those in Adamawa. This can be explained by the fact that a large majority of farmers involved in agriculture in the North Cameroon region are cotton farmers, generally assisted by SODECOTON (Société de Développement du Coton du Cameroun) field agents who ensure that the farmers in their network are well equipped and informed. This conclusion is comparable to those of Ndwata et al. [85] who mentioned that, in the case of Chemba and Kondoa districts in Tanzania, the knowledge of aflatoxin by stakeholder farmers is largely attributed to field schools and training organized by agricultural extension agents. However, no association was established between the practice scores obtained and the production zone, despite the positive association that exists in relation to the knowledge scores, which implies that having better knowledge does not automatically imply having better practice. This can be explained by poverty, the tendency to self-consumption, and the many technical and social constraints faced by farmers. In these situations, farmers favor practices aimed at increasing yield to the detriment of practices that ensure better quality [56].

## 5. Conclusion

The outcome of this study, carried out among smallholders in groundnut-producing communities in Cameroon, showed that farmers' awareness and knowledge of mycotoxins is very low and that they are not at all aware that groundnuts are contaminated by aflatoxins. This lack of awareness and knowledge has the potential to negatively affect their postharvest management methods, through the use of suboptimal practices. The obvious result of such a situation will be higher risks of aflatoxin contamination of groundnut production. On this basis, there is a potential for exposure of the populations consuming the groundnuts supplied by these farmers, which constitutes an obstacle to achieving food security goals. Improving groundnut farmers' access to knowledge on mycotoxins and aflatoxins specifically, and strengthening their knowledge and their capacities in terms of managing mould and aflatoxin contamination are the major challenges imperative for the Cameroonian government. For this purpose of ensuring aflatoxin-free groundnut production by smallholders, it is recommended that food and feed safety authorities raise awareness of mycotoxin issues among groundnut farmers. Extension services and awareness campaigns in this case are crucial. These interventions, aimed at reducing people's exposure to aflatoxin-contaminated grains and ensuring public health, must take into account the sociotechnical aspects of groundnut production.

## Figures and Tables

**Table 1:** Bioclimatic characteristics of the survey regions.

Region	AEZ	Altitude (m)	Precipitation^a^ (mm)	Temperature^b^ (°C)
Adamawa	AEZ 2	400–2000	600–1500	20–32
Centre	AEZ 5	400–1000	1500–2500	23–29
North	AEZ 1	300–1400	400–1200	28–45

AEZ: agroecological zone; source: [22, 28, 29]. ^a^Mean annual precipitations. ^b^Mean annual temperatures.

**Table 2:** Sociodemographic characteristics of groundnut farmers.

Sociodemographic characteristics	Overall, *N* = 380^1^	Adamawa, *N* = 99^1^	Centre, *N* = 161^1^	North, *N* = 120^1^	*χ* ^2^ value	*p* value
Age (years)					21.848	0.001^2^
18–30	81 (21.5%)	26 (26.3%)	18 (11.2%)	37 (31.6%)		
31–45	181 (48.0%)	50 (50.5%)	82 (50.9%)	49 (41.9%)		
46–60	81 (21.5%)	18 (18.2%)	42 (26.1%)	21 (17.9%)		
>60	34 (9.0%)	5 (5.1%)	19 (11.8%)	10 (8.5%)		
Gender					202.43	<0.001^2^
Female	191 (50.3%)	13 (13.1%)	149 (92.5%)	29 (24.2%)		
Male	189 (49.7%)	86 (86.9%)	12 (7.5%)	91 (75.8%)		
Marital status					—	0.041^3^
Single	39 (10.3%)	4 (4.0%)	19 (11.8%)	16 (13.3%)		
Divorced	2 (0.5%)	0 (0.0%)	2 (1.2%)	0 (0.0%)		
Married	339 (89.2%)	95 (96.0%)	140 (87.0%)	104 (86.7%)		
Education level					—	<0.001^3^
Not in school	83 (22.4%)	33 (33.7%)	9 (5.8%)	41 (35.0%)		
Primary	112 (30.3%)	26 (26.5%)	54 (34.8%)	32 (27.4%)		
Secondary	174 (47.0%)	39 (39.8%)	92 (59.4%)	43 (36.8%)		
University	1 (0.3%)	0 (0.0%)	0 (0.0%)	1 (0.9%)		
Land area under groundnuts (ha)					—	<0.001^3^
0–0.5	214 (56.3%)	29 (29.3%)	130 (80.7%)	55 (45.8%)		
0.6–1.0	96 (25.3%)	30 (30.3%)	26 (16.1%)	40 (33.3%)		
1.1–3	65 (17.1%)	37 (37.4%)	4 (2.5%)	24 (20.0%)		
>3	5 (1.3%)	3 (3.0%)	1 (0.6%)	1 (0.8%)		
Average income from groundnut production (FCFA)	636 000	1 508 011	183 684	507 123	218.36	<0.001^4^

^1^ *n* (%), ^2^Pearson's chi-squared test, ^3^Fisher's exact test, ^4^Kruskal–Wallis rank sum test.

**Table 3:** Postharvest practices of groundnut farmers.

Postharvest practices	Overall, *N* = 380^1^	Adamawa, *N* = 99^1^	Centre, *N* = 161^1^	North, *N* = 120^1^	*χ* ^2^ value	*p* value
Drying pods after harvesting	380 (100%)	99 (100%)	161 (100%)	120 (100%)	—	—
Duration of pod drying					156.3	<0.001^2^
0–7 days	220 (59%)	35 (37%)	151 (94%)	34 (29%)		
8–14 days	112 (30%)	36 (38%)	8 (5%)	68 (57%)		
Above 14 days	43 (11%)	24 (25%)	2 (1.2%)	17 (14%)		
Sorting	329 (87%)	90 (91%)	155 (96%)	84 (70%)	13.13	<0.001^2^
Drying location					—	0.012^3^
Home	363 (96%)	94 (95%)	159 (99%)	110 (92%)		
Field	17 (4.5%)	5 (5.1%)	2 (1.2%)	10 (8.3%)		
Drying method					—	<0.001^3^
In the sun with dryer	45 (12%)	1 (1.0%)	44 (27%)	0 (0%)		
In the sun without dryer	323 (87%)	96 (97%)	117 (73%)	110 (98%)		
Others	2 (0.5%)	2 (2.0%)	0 (0%)	0 (0%)		
Under shade	2 (0.5%)	0 (0%)	0 (0%)	2 (1.8%)		
Drying surface					41.86	<0.001^2^
On the ground	214 (58%)	73 (74%)	75 (47%)	66 (59%)		
Cemented area	23 (6.2%)	0 (0%)	23 (14%)	0 (0%)		
On a tarpaulin	135 (36%)	26 (26%)	63 (39%)	46 (41%)		
Good drying indicator						
Sound of seed in pod	322 (85%)	95 (96%)	131 (81%)	96 (80%)	13.13	0.001^2^
Pod cracks easily	156 (41%)	12 (12%)	125 (78%)	19 (16%)	154.8	<0.001^2^
Other method	13 (3.4%)	0 (0%)	13 (8.1%)	0 (0%)	—	<0.001^3^
Using a moisture meter	0 (0%)	—	—	—	—	—
Grain deterioration indicator						
Color other than that typical of the seed	291 (77%)	91 (92%)	118 (73%)	82 (68%)	18.50	<0.001^2^
Taste (Bitter)	109 (29%)	23 (23%)	64 (40%)	22 (18%)	17.36	<0.001^2^
No insect damage	109 (29%)	10 (10%)	68 (42%)	31 (26%)	31.64	<0.001^2^
Others	49 (13%)	13 (13%)	23 (14%)	13 (11%)	0.73	0.7^2^
Duration of storage in pods					—	<0.001^3^
0–3 months	52 (16%)	6 (7.3%)	44 (35%)	2 (1.7%)		
3–6 months	49 (15%)	10 (12%)	15 (12%)	24 (20%)		
>6 months	224 (69%)	66 (80%)	66 (53%)	92 (78%)		
Type of bags used for storage					—	<0.001^3^
Polypropylene bags	273 (75%)	85 (99%)	87 (54%)	101 (86%)		
Jute bag	19 (5.2%)	0 (0%)	5 (3.1%)	14 (12%)		
Polyethylene bags	50 (14%)	1 (1.2%)	49 (31%)	0 (0%)		
Hermetic bag	17 (4.7%)	0 (0%)	17 (11%)	0 (0%)		
Others	4 (1.1%)	0 (0%)	2 (1.3%)	2 (1.7%)		
Threshing method used					257.4	<0.001^2^
Hand threshing	258 (68%)	29 (29%)	161 (100%)	68 (57%)		
Use thresher	86 (23%)	69 (70%)	0 (0%)	17 (14%)		
Both	36 (9%)	1 (1.0%)	0 (0%)	35 (29%)		
Practice scores					—	0.1784^3^
Poor (0–7)	375 (98.7%)	98 (99%)	157 (98%)	120 (100%)		
Good (8–12)	5 (1.3%)	1 (1.0%)	4 (2.5%)	0 (0%)		

^1^ *n* (%), ^2^Pearson's chi-squared test, ^3^Fisher's exact test.

**Table 4:** Groundnut farmers' knowledge of mycotoxins and aflatoxins in the Adamawa, Centre, and North Cameroon regions.

Knowledge of mycotoxins and aflatoxins	Overall, *N* = 380^1^	Adamawa, *N* = 99^1^	Centre, *N* = 161^1^	North, *N* = 120^1^	*χ* ^2^ value	*p* value
Are you aware of mycotoxins in groundnuts? Yes	45 (12%)	8 (8.1%)	35 (22%)	2 (1.7%)	28.34	<0.001^2^
Are you aware of aflatoxins in groundnuts? Yes	0 (0%)	—	—	—	—	—
Are you aware of the link between mould contamination and mycotoxin contamination in groundnuts? Yes	27 (7.1%)	7 (7.1%)	18 (11%)	2 (1.7%)	9.42	0.009^2^
Are you aware of the effects of mycotoxins on consumer health? Yes	26 (7%)	7 (7.2%)	17 (11%)	2 (1.8%)	7.86	0.020^2^
Knowledge scores					2.43	0.3^2^
(0, 10) poor	325 (86%)	88 (89%)	139 (86%)	98 (82%)		
(10, 16) good	55 (14%)	11 (11%)	22 (14%)	22 (18%)		

^1^ *n* (%), ^2^Pearson's chi-squared test, ^3^Fisher's exact test.

**Table 5:** Socioeconomic characteristics associated with knowledge of mycotoxin contamination of groundnuts and use of mould-contaminated grain as a food source in the Adamawa, Centre, and North Cameroon regions.

Socioeconomic characteristics	Knowledge of mycotoxins		Use of mould-contaminated grain as a food source	
	OR^1^ (95% CI^1^)	*p* value	OR^1^ (95% CI^1^)	*p* value
Age (years)				
18–30	—		—	
31–45	1.03 (0.37–3.19)	>0.9	2.10 (1.02, 4.16)	0.029
46–60	1.55 (0.46–5.59)	0.5	2.60 (1.14, 6.02)	0.026
>60	1.34 (0.23–7.12)	0.7	3.34 (1.14, 10.2)	0.03
Gender				
Female	—		—	
Male	1.21 (0.34, 4.03)	0.8	0.43 (0.26, 1.05)	0.026
Marital status				
Single	—		—	
Married	1.20 (0.39, 4.57)	0.8	0.35 (0.14, 0.79)	0.015
Education level				
No study	—		—	
Primary	2.29 (0.63, 11.1)	0.2	1.12 (0.56, 2.21)	0.8
Secondary	3.42 (1.00, 16.0)	0.043	1.71 (0.86, 3.44)	0.13
University	0.00	>0.9	0.00	>0.9
Experience in culture (years)				
(0, 11)	—		—	
(11, 21)	1.44 (0.59, 3.51)	0.4	0.59 (0.33, 1.06)	0.078
>20	1.59 (0.62, 4.04)	0.3	0.68 (0.37, 1.24)	0.2
Region				
Adamawa	—		—	
Centre	4.49 (1.11, 21.0)	0.044	0.39 (0.15, 1.00)	0.055
North	0.32 (0.05, 1.47)	0.2	0.27 (0.13, 0.56)	<0.001

^1^OR: odds ratio; CI: confidence interval.

**Table 6:** Sociodemographic factors associated with scores for knowledge and practices concerning the management of kernel contamination by moulds and mycotoxins.

Socioeconomic characteristics	Knowledge scores (<10)		Practice scores (<7)	
	OR^1^ (95% CI^1^)	*p* value	OR^1^ (95% CI^1^)	*p* value
Age (years)				
18–30	—		—	
31–45	0.38 (0.16, 0.87)	0.023	0.42 (0.10, 1.99)	0.3
46–60	0.63 (0.46, 1.76)	0.4	0.33 (0.05, 2.60)	0.3
>60	0.23 (0.03, 1.11)	0.094	0.44 (0.04, 3.94)	0.5
Gender				
Female	—			
Male	0.53 (0.20, 1.44)	0.2	0.15 (0.20, 1.44)	0.057
Marital status				
Single	—			
Married	1.00 (0.38, 2.88)	>0.9	0.91 (0.18, 7.5)	>0.9
Education level				
No study	—			
Primary	1.22 (0.45, 3.45)	0.7	1.26 (0.24, 7.59)	0.8
Secondary	1.32 (0.52, 3.60)	0.6	1.27 (0.26, 7.82)	0.6
University	0.00	>0.9	0.00	>0.9
Experience in culture (years)				
(0, 11)	—		—	
(11, 21)	0.66 (0.59, 1.51)	0.31	3.80 (0.85, 21.3)	0.09
>20	1.35 (0.60, 3.06)	0.5	6.06 (0.60, 38.0)	0.03
Region				
Adamawa	—		—	
Centre	1.43 (0.41, 5.38)	0.6	0.89 (0.10, 6.61)	>0.9
North	3.31 (0.05, 1.47)	0.015	0.87 (0.12, 8.77)	0.9

^1^OR: odds ratio; CI: confidence interval.

## Data Availability

The datasets generated during and/or analyzed during the current study are available from the corresponding author upon reasonable request.
